# Effect of *Rudbeckia laciniata* invasion on soil seed banks of different types of meadow communities

**DOI:** 10.1038/s41598-022-14681-1

**Published:** 2022-06-29

**Authors:** Elżbieta Jędrzejczak, Ewelina Klichowska, Marcin Nobis

**Affiliations:** Institute of Botany, Faculty of Biology, Jagiellonian University, Gronostajowa 3, 30-387 Kraków, Poland

**Keywords:** Plant ecology, Invasive species

## Abstract

In the last decades, biological invasions become the main driver of biodiversity loss. The changes can be noticed not only in the above-ground diversity but also in the underground, including seed banks of native vegetation. In this study, we focus on *Rudbeckia laciniata*, a species introduced to many European and Asian countries, to characterize its soil seed bank as well as to answer the question, how the species influenced soil seed banks of meadow plant communities in two types of habitats (fresh and wet), where traditional mowing was abandoned. Within the habitats, we conducted our study on a three-step scale of invasion, from full invasion, through the transition zone to the control zone, where no invasion of the species has been observed so far. The majority of the *R. laciniata* seeds were located in the surface layer of soil. We detected, that 47% (in fresh meadow) and 56% (wet meadow) of recorded species occurred only in a soil seed bank, and were absent in aboveground vegetation. Emergence of native plants from the soil seed bank is low due to rapid shading of the soil surface by *R. laciniata* seedlings. However, a short-term seed bank of the species gives hope that returning to regular mowing brings the desired results in its the elimination from vegetation, in a fairly short time.

## Introduction

Invasive species are one of the main factors changing the environment on a global scale^1–3^. Many of these organisms have led to economic losses in agriculture, forestry and fisheries^3,4^, and the prevention, control and preparation of effective invasive species management plans has therefore become a priority. Monospecific patches of invasive plants in meadows, unused arable fields, along roadsides, or even in river valleys have become common, therefore many studies are investigating the effects of plant invasions on above-ground vegetation^5–7^. For instance, in recent years, increasing numbers of reports have emerged on the negative impact of invasive species on soil microbial communities^7–9^ as well as soil seed banks^10–13^.

Seed banks are reservoirs of seeds in the soil. They reflect not only the present but also the past vegetation, and depending on the persistence of the seeds, they reflect the potential of the community for regeneration^14,15^. According to Gioria and Pyšek^15^, the most important aspects connected with invasive species, such as the potential of their persistence in a community, the community’s ability to buffer the displacement of native species from the vegetation, as well as changes in the biotic and abiotic conditions associated with an invasion are strongly linked to the properties of the seed bank of native and alien species. Changes in the species composition of the seed banks of communities affected by the invasion take place more slowly than in the aboveground communities^11,15,16^. Only when the species disappears from both the above-ground vegetation and the seed bank, their loss is no longer reversible without new introductions or a dispersal event^15,17,18^. Thus, the actual regeneration capacity of a community can only be assessed on the basis of knowledge about the species composition of the seed bank. Another important aspect for the prevention, control and management of invasions is understanding the reproductive biology of the invasive species, including seed production, germination or the persistence of their seeds in the soil seed banks^15,18,19^. As propagule and genetic diversity reservoirs, soil seed banks should be considered a major factor affecting invasion dynamics^15,18,20,21^. The ability to form persistent seed banks might determine the successful establishment and potential for the invasiveness of alien species in a new geographic range, as well as their persistence under different environmental conditions. As a result, this factor can decide which introduced species will succeed^15,18,21,22^. Moreover, some studies show that alien species of plants can influence the composition of native species in seed banks, even if they reproduce only vegetatively^23^. An indirect influence on soil seed banks occurs through the changes in the abiotic and biotic conditions of habitats, by blocking the inflow of new diasporas, shading^11^, as well as stimulating native species to vegetative reproduction by increasing environmental stress^18^. On the other hand, the impact of invasive species on soil seed banks can vary significantly depending on the above-ground vegetation^11,12,24^ and type of habitat^25^.

Despite growing interest among researchers, the impact of invasive plant species on soil seed banks is still identified as an urgent research priority^26^. This is especially true in the case of species whose invasive potential was so far underestimated, such as *Rudbeckia laciniata* (Asteraceae). This species is native to central and eastern North America and was introduced to Europe in the seventeenth century as an ornamental plant. The first information on the occurrence of *R. laciniata* outside of cultivation comes from the end of eighteenth century^27,28^, and today, the species has been reported in many European countries^4,29–31^ as well as in Asian part of Russia, China, Japan and New Zealand^27,32–34^. Despite its ability to spread beyond its natural range and a tendency to quickly expand^19^, the negative impact of this species on vegetation has long been overlooked. There are also no documented studies on the influence of *Rudbeckia* on soil seed banks, and studies referring to the reproductive abilities of this species provide inconsistent results^35–37^. Thus in this study, we investigate the impact of *R. laciniata* on the soil seed banks of meadow plant communities in two different types of habitats (wet and fresh meadows), where traditional mowing has been abandoned. We would like to answer the following questions: (1) how does the species composition of the seed bank differ from the species composition of the above-ground vegetation?; (2) does the abundance and species composition of the soil seed bank vary significantly along the gradient of the *R. laciniata* invasion and (3) what is the vertical distribution of *R. laciniata* seed bank in soil?

## Materials and methods

### Study species

*Rudbeckia laciniata* (cutleaf coneflower), is a perennial plant species from the family Asteraceae, growing up to 50–300 cm. It has broadly ovate leaves (8–40 × 3–20 cm), 1–2-pinnatifid or pinnately compound with 3–11 leaflets/lobes, usually glabrous, petiolate or sessile. The flower heads has 5–10 cm in diameter, and they are arranged in loose, corymbiform arrays. In each head there are 8–12, laminae elliptic to oblanceolate, golden-yellow, barren, ray flowers as well as 150–300 + yellow to yellowish-green, disc flowers with stamen and pistil. Cypselae is 3–4.5 mm long with pappi coroniform or of 4 scales, up to 1.5 mm long. It blooms from August to October^38^.

*Rudbeckia laciniata* prefers moist habitats, rivers, streams, and drainage ditches banks. It also grows in ruderal habitats, along roads and in gardens^38^.

Common in Europe, *R. laciniata* var. *laciniata* is characterized by a relatively high proportion of apomictic reproduction. Francírková^35^ reports that *Rudbeckia laciniata* successfully reproduces vegetatively even from fluff fragments only one centimeter long. In Central Europe, typical variety has 2n = 64–76, while the double-flowered variety "Golden Glow", 2n = 38^27^.

### Study area

**Figure 1:**
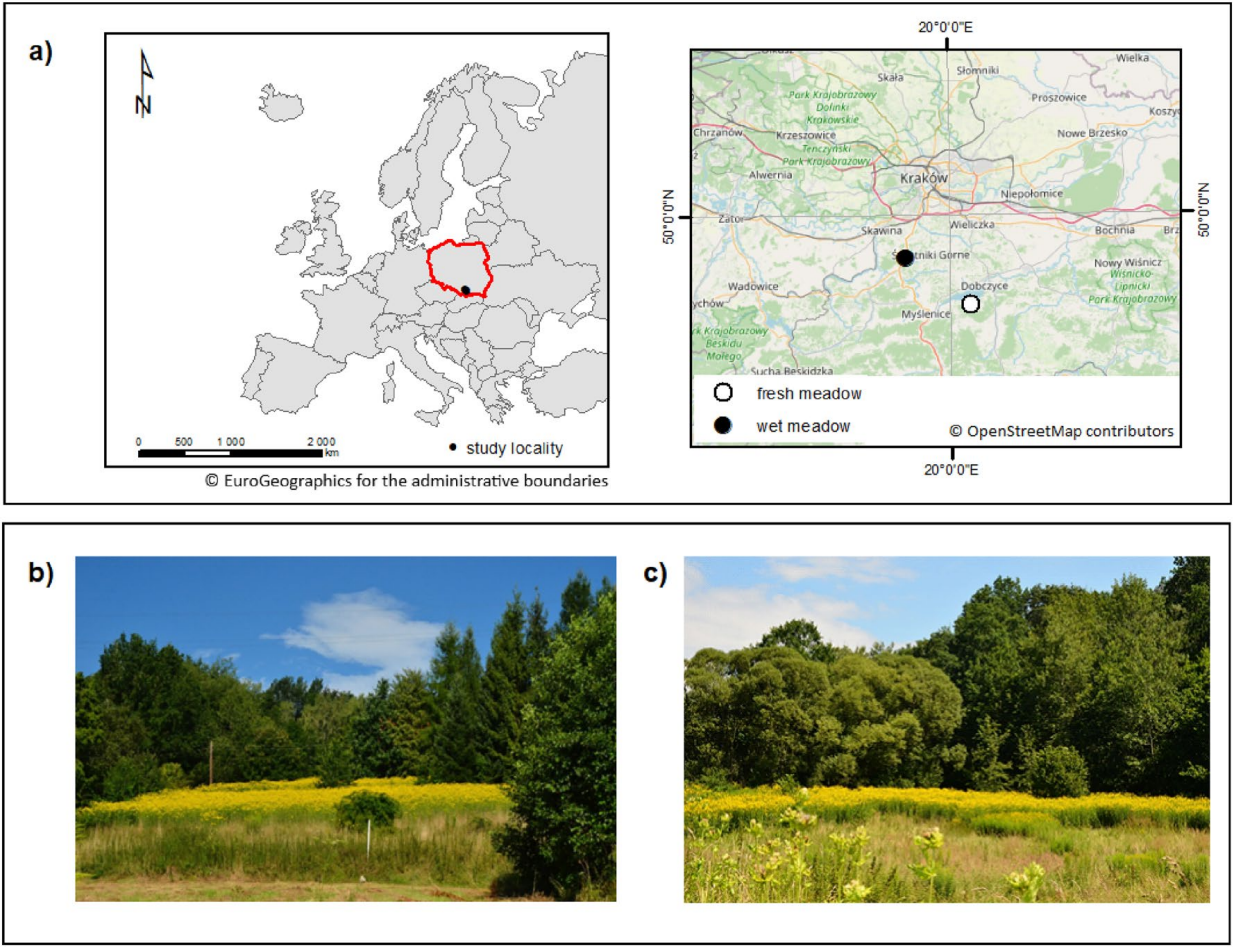
Study area. Location (**a**) and a view on the investigate plots: fresh (**b**) and wet (**c**) meadow. Yellow patches is *Rudbeckia laciniata*. Layer of European countries from Eurostat, topographic basemap from OpenStreetMap https://www.openstreetmap.org/copyright.

The study was conducted in southern Poland in two types of abandoned meadows. The first location was outside a river valley in a fresh meadow (Kornatka village, N 49.848906, E 20.054445) and the second was within a river valley in a wet meadow (Mogilany village, N 49.928107, E 19.886026) (Fig. 1). The locations met the following conditions: the occurrence of a coherent plot of an invasive species of an area equal to 0.5 ha, no agriculture or any other types of land use (mowing, grazing, burning) within the *Rudbeckia* plots, population over 10 years old, and the presence of a sizeable enough control area lacking *R. laciniata* in the immediate vicinity. Three zones were designated within the borders of both experimental plots: A—full invasion, where *R. laciniata* forms a compact patch of vegetation, the abundance of the invasive species exceeds 70% of the vegetation cover; B—intermediate invasion, *R. laciniata* occurs with medium cover (less than 70% cover but greater than 0% cover), and zone C—control, where *R. laciniata* was not reported, but due to the direct proximity and similar topography was most likely to represent the same type of vegetation cover as in zone A before the invasion.

### Sampling and soil seed bank estimating

Within each zone, 125 soil samples were taken with a 5-cm diameter cylinder. Each sample was divided into an upper layer (0–5 cm) and a lower layer (5–10 cm). Samples within one category were combined randomly so that a total of 20 samples from the upper and lower layers from each zone were prepared. Soil samples were taken early in spring, before the beginning of the vegetation season, so that seeds could go through natural winter stratification. Soil samples were cleaned from the fragments of rhizomes and stones and put into plastic pots. The pots were previously filled with a blend of sterile sand and garden soil. Then a thin (about 3–4 cm) layer of the soil sample taken in the field was put on top. The medium was prepared in this way to facilitate the rooting of germinating seedlings. Additionally, to secure the sterility of the medium, six control pots were prepared with the mixture of garden soil and sand, but without the layer of soil taken from the field. Control pots were randomly distributed among the pots with soil taken from the field samples. All the pots were placed under laboratory conditions. The growth of the seedlings was checked twice a week. The seedlings were kept in pots until the time when species identification was possible. Then they were removed to minimize competition with new seedlings. Some specimens could be identified only to the level of genus and in the case of monocotyledons—to the level of family. When there were no seedlings in the pot, the upper layer of soil was delicately mixed. This measure was taken to stop seed dormancy and stimulate their germination^20,39^.

The study of the soil seed bank was conducted using the seedling emergence method. In the case of soil seed banks, the number of seedlings and the abundance of the soil seed bank were calculated. The abundance of soil seed bank was estimated on the basis of the number of seedlings and expressed as the number of seeds per 1 m^2^ (seeds/m^2^).

Before starting the research on the soil seed bank, 100 plots of vegetation of an area of 1 m^2^ were designated within each study zone. The following five-degree scale (1—1–5%; 2—5–25%; 3—25–50%; 4—50–75%; 5—75–100%) was used to determine plant cover-abundance on each plot.

### Statistical analyses

Basic biodiversity indexes were calculated, including the number of taxa in the studied zones, richness of species (number of species in particular plots), Shannon–Wiener diversity index and dominance. In addition, the average number of species per plot/sample with the standard deviation was determined. Differences in mean biodiversity indexes, as well as in the composition of species between subsequent zones (A, B and C) were tested with the one-way PERMANOVA, and between plots (fresh and wet meadow) with the two-way PERMANOVA. Additionally, the SIPMER analysis was performed, to indicate which species had the greatest impact on the differences between the studied invasion zones. In the case of *R. laciniata*, the sum of seedlings and mean number of seedlings in a sample with the standard deviation were also determined.

The assessment of the similarity between the vegetation cover and soil seed bank was based on the calculation of the Sorensen index for binary data (without the quantitative participation of species) and the set of percentage participation of taxa occurring: (1) in the soil seed bank, (2) in the vegetation cover and (3) common for the seed bank and the vegetation cover.

## Results

### Soil seed bank under pressure of *R. laciniata* invasion

The total numbers of species found in the soil seed bank in subsequent invasion zones were similar in both examined types of meadows. The highest number of species was found in the transition zone (B) in the wet meadow (56 species), whereas the smallest number was in the transition zone in the fresh meadow (43 species). The total number of species in the fresh meadow ranged between 43 in zone B and 49 in zone C, while in the wet meadow, it was between 46 and 55 species in zones A and B, respectively (Table 1).

**Table 1:** The abundance of the soil seed bank and diversity indexes in three studied zones and two habitats (fresh and wet meadow).

Habitat type	Zone	∑	${\overline{\text{X}}}$	The abundance of the seed bank	Number of species	Richness of species	Diversity	Dominance
Fresh	A	1890	94	7560	44	9.08	1.86	0.33
	B	986	49	3944	43	9.03	2.55	0.13
	C	1135	57	4540	49	9.78	2.65	0.12
Wet	A	5282	264	21,128	46	9.55	0.91	0.69
	B	1319	66	5276	56	11.80	2.73	0.10
	C	1251	63	5004	53	10.60	2.68	0.12

*A* invasion zone, *B* transition zone, *C* control zone, *∑* total number of seedlings, ${\overline{\text{X}}}$ mean number of seedlings in a sample (upper layer plus lower layer of soil); *the abundance of the seed bank* (seeds)/m^2^, *Number of species* total number of seedlings’ species detected in a given zone, * species richness* mean number of seedling species in a sample, *Diversity* mean Shannon–Wiener index in a sample, *Dominance* mean index of value 1 minus Simpson index in a sample.

The one-way PERMANOVA indicated the lack of differences between the species richness in the soil seed bank between subsequent zones in the fresh meadow. In the wet meadow, the differences in species richness were only observed between the invasion zone (A) and the transition zone (B). Significant differences were observed in the number of seedlings. In the fresh meadow, significant differences were observed between all zones, while in the wet meadow, no differences between the transition and control zone were observed (Table 2). The abundance of the soil seed bank in both habitats was greater in the invasion zone than in the two remaining ones. In the fresh meadow, the abundance of the soil seed bank was 7560 and 4540 seeds/m^2^, whereas in the wet meadow, 21,128 and 5004 seeds/m^2^ in the invasion and control zones, respectively. In both studied habitats, the dominance index in the invasion zone was the highest and it significantly differed from the values in the other zones, whereas the highest diversity was observed in the transition and control zones (Table 1). The two-way PERMANOVA showed significant differences in soil seed bank for both, ‘habitat type’ and ‘zone’ factors, as well as a significant effect of ‘habitat type × zone’ interaction for each tested index (Table 3). In the fresh meadow, *R. laciniata* contribution to the dissimilarity between studied zones was 35% and it was almost two times lower than in the case of wet meadow (60%) (Table 4). In the fresh meadow, *Holcus lanatus* greatly contributed to the dissimilarity between zones, with a large average abundance in the control zone and small participation in other zones. In the wet meadow, *Urtica dioica, Juncus *sp. and *Hypericum maculatum* also greatly contributed to the dissimilarity between zones (Table 4).

**Table 2:** Results of one-way PERMANOVA for the soil seed bank; differences in the number of seedlings and in diversity indexes between zones.

	TSS	SS	F	p	A–B	B–C	A–C
**Fresh**							
Number of seedlings	1.52	0.54	51.51*	0.0001	+	+	+
Species composition	10.45	3.67	52.75*	0.0001	+	+	+
Richness of species	0.55	0.51	2.28	0.1082	−	−	−
Diversity	0.65	0.28	37.59*	0.0001	+	−	+
Dominance	2.86	1.05	49.05*	0.0001	+	−	+
**Wet**							
Number of seedlings	5.46	0.73	185.80*	0.0001	+	−	+
Species composition	13.01	3.77	69.93*	0.0001	+	+	+
Richness of species	0.51	0.41	7.13*	0.0019	+	−	−
Diversity	3.30	0.29	296.60*	0.0001	+	−	+
Dominance	6.42	0.93	168.30*	0.0001	+	+	+

*TSS* total sums of square, *SS* sums of squares, *F* F value, *p* p value, *A* invasion zone, *B* transition zone, *C* control zone, − no difference (p ≥ 0.05); + significant differences (p < 0.05); *statistically significant result (p < 0.05).

**Table 3:** Results of two-way PERMANOVA for the soil seed bank; differences in the number of seedlings and diversity indexes for zones and habitat types.

	SS	df	MS	F	p
**Number of seedlings**					
Habitat type	1.0589	1	1.06	95.146*	0.0001
Zone	4.393	2	2.2	197.36*	0.0001
Habitat type × zone	1.323	2	0.66	59.438*	0.0001
**Species composition**					
Habitat type	5.09	1	5.09	78.08*	0.0001
Zone	11.67	2	5.84	89.51*	0.0001
Habitat type × zone	4.36	2	2.18	33.40*	0.0001
**Richness of species**					
Habitat type	0.04	1	0.04	5.48*	0.0176
Zone	0.08	2	0.04	5.16*	0.0066
Habitat type × zone	0.06	2	0.03	3.69*	0.0276
**Diversity**					
Habitat type	0.32	1	0.32	64.58*	0.0001
Zone	2.65	2	1.33	266.57*	0.0001
Habitat type × zone	0.72	2	0.36	72.58*	0.0001
**Dominance**					
Habitat type	0.4	1	0.4	22.79*	0.0001
Zone	6.55	2	3.28	188.47*	0.0001
Habitat type × zone	0.75	2	0.37	21.54*	0.0001

*SS* sums of squares, *df* number of degrees of freedom, *MS* mean square, *F* value F, *p* value p; *statistically significant result (p < 0.05).

**Table 4:** Results of SIMPER analysis.

Species	Contribution (%)	Cumulative contribution (%)	Average abundance		
			Zone A	Zone B	Zone C
**Fresh**					
*Rudbeckia laciniata*	34.56	34.56	51.6	2	0
*Holcus lanatus*	11.91	46.47	2.05	2.75	16.3
*Trifolium repens*	9.978	56.45	1.85	12.8	0.55
*Urtica dioica*	7.789	64.24	13.3	3.95	5.35
*Chenopodium polyspermum*	6.334	70.57	3.8	9.7	5.3
*Stellaria graminea*	3.372	73.94	3.85	2.8	4.55
*Ranunculus repens*	2.989	76.93	2.45	2.3	4.85
**Wet**					
*Rudbeckia laciniata*	60.41	60.41	218	6.8	0
*Urtica dioica*	5.825	66.23	12.6	13.1	16.1
*Juncus sp.*	4.333	70.57	8	10.9	3.05
*Hypericum maculatum*	3.742	74.31	1.65	4.9	7.75
*Chenopodium polyspermum*	3.015	77.32	1.2	7.2	5.1

Contribution of the most important species present in the soil seed bank to the dissimilarity between the subsequent zones in the fresh and in the wet meadow.

The abundance of Rudbeckia's soil seed bank was significantly greater in the invasion zone than in the transition zone. The control zones had no seeds of this species. In the wet meadow, the bank of *R. laciniata* in the invasion zone was 17,476 seeds/m^2^, and in the fresh meadow, it was 4132 seeds/m^2^ (Table 5). In the transition zones, there were 160 and 544 seeds/m^2^ in the fresh and wet meadow, respectively. Both in the fresh and wet meadows, the majority of *R. laciniata* seeds were located in the upper layer of soil (0–5 cm) (Table 5).

**Table 5:** The abundance of *Rudbeckia laciniata* soil seed bank in studied zones.

Habitat type	Zone	Layer	Abundance of soil seed bank	∑	${\overline{\text{X}}}$	SD	fr. [%]
Fresh	A	Upper	4132	1001	50.05	18.25	100
		Lower		32	1.60	1.50	75
	B	Upper	160	36	1.80	2.65	60
		Lower		4	0.20	0.41	20
	C	Upper	–	–	–	–	–
		Lower		–	–	–	–
Total			1431	1073			
Wet	A	Upper	17,476	4005	202.75	41.22	100
		Lower		314	15.70	6.74	100
	B	Upper	544	129	6.45	2.96	100
		Lower		7	0.35	0.67	25
	C	Upper	–	–	–	–	–
		Lower		–	–	–	–
Total			6007	4455			

*A* invasion zone, *B* transition zone, *C* control zone, *upper* layer of soil 0–5 cm, *lower* layer of soil 5–10 cm; Abundance of the seed bank—number of seeds *R. laciniata* /m^2^, *∑ *total number of seedlings, ${\overline{\text{X}}}$ mean number of seedlings in a sample, *SD* standard deviation, *fr* frequency (percentage of pots with seedlings per 20 pots).

**Figure 2:**
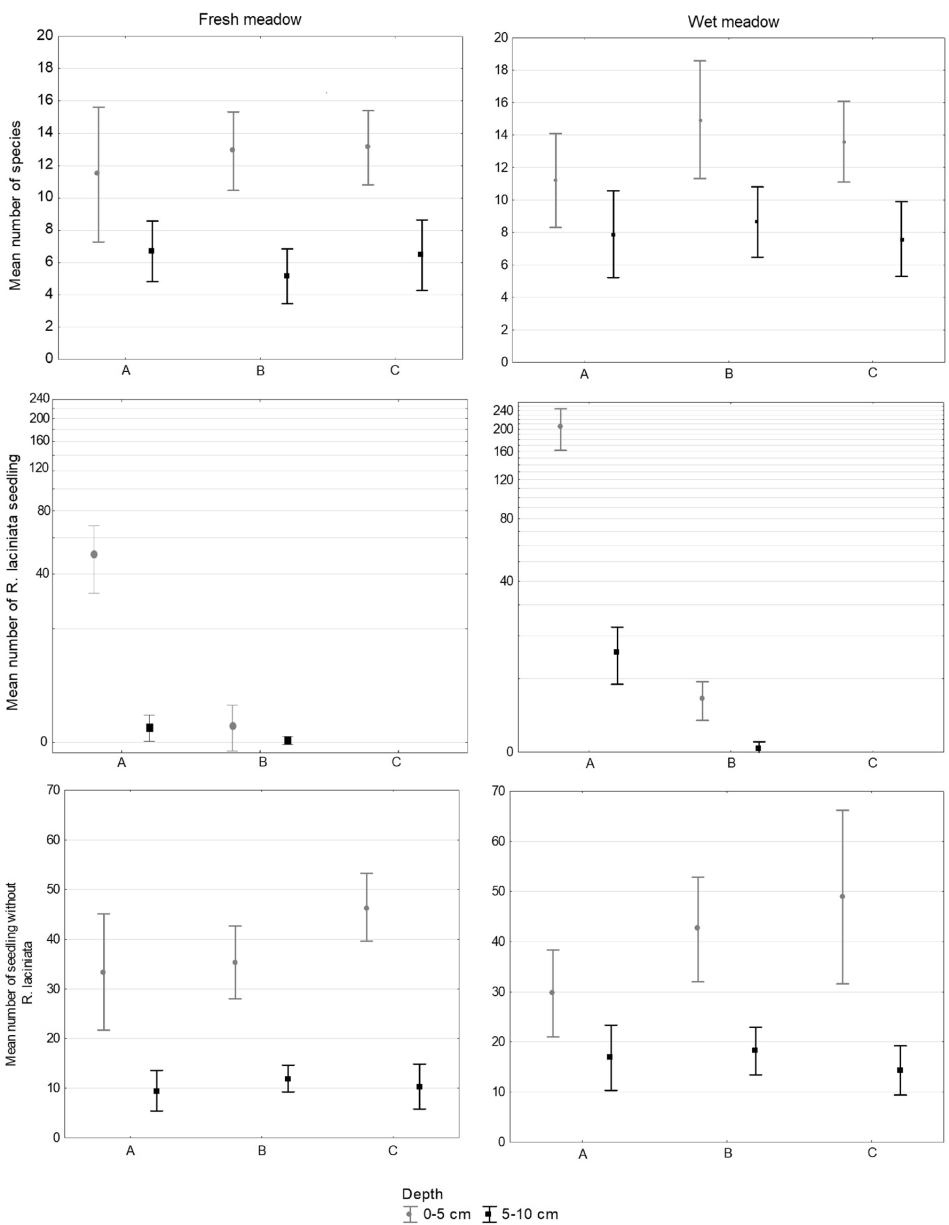
Mean number of species and seedlings with the standard deviation in each zones (A—invasion, B—transition, C—control) and depth layer (grey: 0–5 cm; black: 5–10 cm) in two types of meadow.

The number of seedlings, excluding *R. laciniata*, was greater in the upper soil layer than in the lower one and was similar for both studied types of meadows. Furthermore, the number of *R. laciniata* seedlings was much higher in the upper soil layer than in the lower one for the invasion zone, and to a lesser extent also for the transition zone, in both examined types of meadows. For both types of meadows, we recorded a greater number of species in the upper than in the lower layer (Fig. 2).

Apart from *Rudbeckia*, the largest soil seed bank in the invasion zone in both studied habitats was formed by *Urtica dioica*, which was followed by *Juncus* sp. (in wet meadow) and Poaceae sp. (in fresh meadow), *Stellaria graminaea* is in third place in the case of fresh meadow, while in third place in the case of wet meadow and fourth in the case of fresh meadow is *Chenopodium polyspermum*. In the transition zone, *Urtica dioica* remains in the first place only in the case of a wet meadow, in a fresh meadow the most abundant seed bank is represented by *Trifolium repens*, followed by *Chenopodium polyspermum* and *Urtica dioica* on the third place, whereas in the fresh meadow the next most abundant seed banks are formed by *Hypericum maculatum,* Poaceae sp. and *Chenopodium polyspermum*. In the wet meadow, also *Solidago canadensis* had a relatively high share in the upper layer of soil in all three studied zones (Supplementary Information S1, S2).

### The dynamics of *Rudbeckia laciniata* seedlings

**Figure 3:**
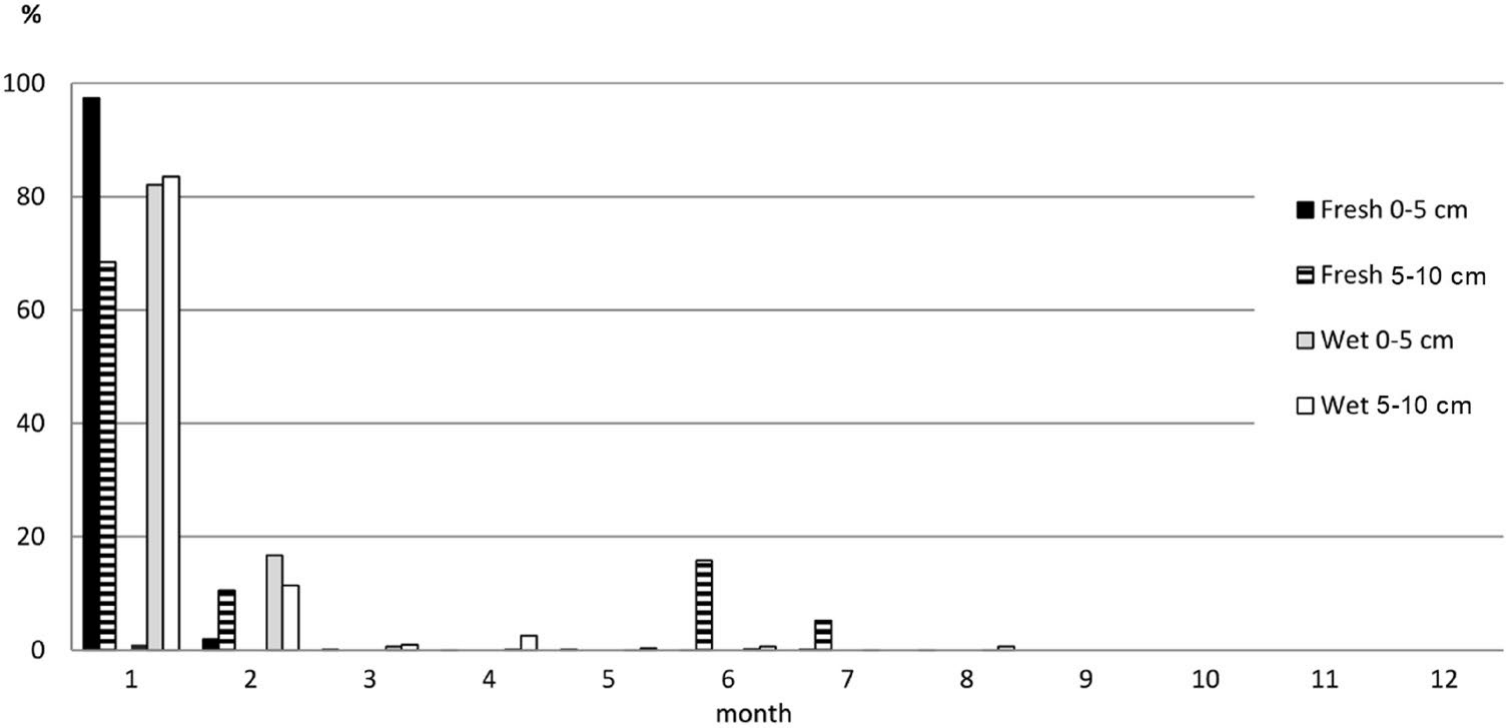
Percentage of *R. laciniata* seedlings appearing in the upper (0–5 cm) and lower (5–10 cm) soil layers in the following months of the experiment.

During subsequent months of observations/experiment, the number of *R. laciniata* seedlings varied considerably. Numerous *Rudbeckia* seedlings appeared rapidly after the start of the experiment. In the period between the second and 30th day of the experiment, more than 80% of the seeds of this species germinated from the upper layer of soil. A similar trend took place in samples from both habitats. No further *Rudbeckia* seedlings were observed as of the 9th month of the experiment (Fig. 3). Of the native species, only single seedlings of *U. dioica, Stellaria graminea* and *Chenopodium polyspermum* started growing in the first week of the experiment. However, the highest number of seedlings of the native plants appeared in the II–V months of the experiment and after removal of the *Rudbeckia* seedlings.

### The relationship between soil seed bank and vegetation cover

The highest similarity between the vegetation cover and soil seed bank was observed in the control zone in the wet meadow (Sorensen index was 0.6) and the smallest in the invasion zones in both habitats, as well as in the control zone in the fresh meadow (0.4). The similarity coefficient in the transition zones was 0.5 for both types of habitats (Supplementary Information S3).

**Figure 4:**
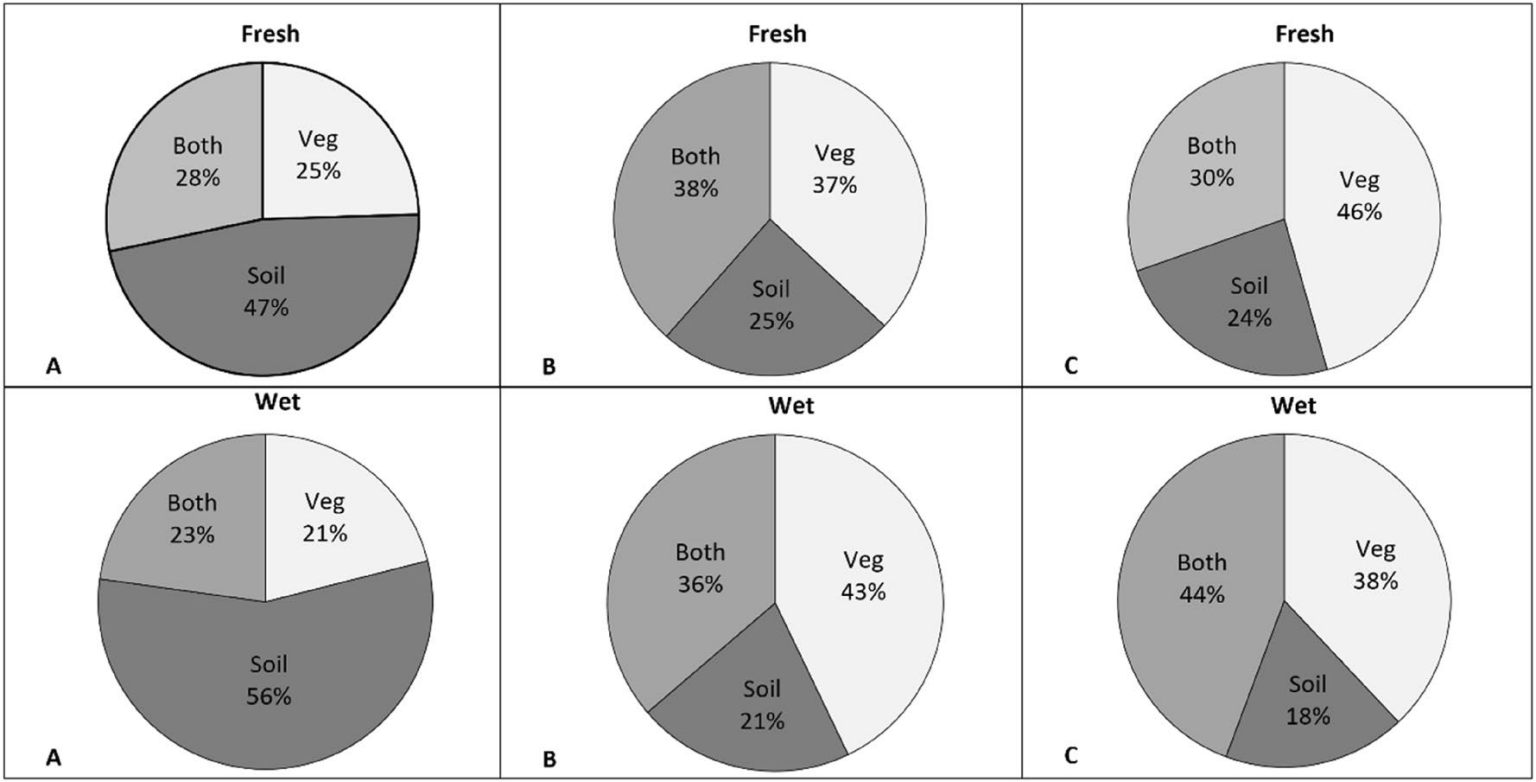
Percentage of the number of taxa occurring in soil seed bank and vegetation cover in the fresh and the wet meadow. (**A**) Invasion zone; (**B**) transition zone; (**C**) control zone; *Veg* participation of taxa occurring only in the ground vegetation, *Soil* participation of taxa occurring only in the soil seed bank, *Both* participation of taxa common for the two groups.

The highest participation of taxa occurring only in the seed bank was observed in the invasion zone (47% in the fresh, 56% in the wet meadow). In other zones, it did not exceed 25%. In the transition zone in both habitat types, the share of taxa common for the two habitat types and the ones occurring only in the vegetation cover increased in comparison to the invasion zone. The control zone in the fresh meadow had the highest share of taxa occurring only in the vegetation (46%), while in the wet meadow, the predominant taxa were the ones common for both, the vegetation cover and the soil seed bank (44%) (Fig. 4, Supplementary Information S4).

## Discussion

### The soil seed bank of *Rudbeckia laciniata*

*Rudbeckia laciniata* created an abundant soil seed bank in both examined meadows. However, the differences in the number of *R. laciniata* seeds in both studied invasion zones (4000 in the fresh vs. 17,000 seed/m^2^ in the wet meadow, respectively) is difficult to explain. Taking into account that the population of the species in both examined meadows are of similar age and that the species makes a short-term bank of seeds (its seeds remain alive in soil for up to 3 years)^40^, the most probable explanation is that the greater production of living seeds by *Rudbeckia* depends on water availability. Humid habitats can positively stimulate the seed production of this species in populations occurring along river and stream valleys^28,41^. On the other hand, higher moisture of this habitat may impact on (1) maintaining seeds viability longer in comparison to the relatively drier conditions of the fresh meadows or/and (2) extending seeds germination in time due to variable environmental conditions (level of water, temperature, etc.) and not leaving the soil seed bank as rapidly as they might in the more stable environmental condition existing within fresh meadows. A similar situation was observed in the case of *Gunnera tinctoria*^42^. Germination can be halted under conditions of increased humidity, which can stimulate the production of an abundant soil seed bank. However, *Gunnera* is a species forming persistent seed banks, thus it can accumulate seeds in the soil, which for various reasons did not germinate immediately after the seeds fell. A high density of seeds on the banks of water bodies was also mentioned by Harper^14^. In studies on germinating *R. laciniata* seeds taken from plants occurring in wet habitats, Francírková^35^ showed that the mean number of living seeds per one individual was about 1600, while only 40% of them germinated in laboratory conditions. In the same paper, the author reports that the average number of *Rudbeckia* seeds per one square meter is about 95,000, which results in approximately 38,000 germinating seeds. This value twice exceeds the abundance of the soil seed bank of *R. laciniata* during the experiment of germinating seeds from the examined wet meadow in Mogilany. However, the number of produced seeds, due to the presence of the environmental ‘sieve’ is always higher than the number of germinating seedlings from the soil seed bank^14^. This can lead to overestimated results in the case of germinating seeds taken directly from parental plants in relation to the germination of seedlings from the soil bank. The author does not give the source of the data referring to the number of specimens of *R. laciniata* in the area of one meter. On the other hand, Moravcová et al.^36^ obtained quite different results—for single individuals, the number of seeds was estimated at 900, which equals about 6500 seeds per 1 m^2^. Moreover, more than 90% of the *Rudbeckia* seeds were able to germinate. It seems that the abundance of the soil seed bank of *R. laciniata* in the examined wet meadow results from more comfortable habitat conditions for this species.

A significantly lower number of seedlings in the transition zones of both examined types of habitats and the lack of seedlings in the control zones prove that the dispersion of *R. laciniata* seeds is spatially limited to several meters. Although Tokarska-Guzik^28^ mentioned anemochory, zoochory and myrmecochory among the ways *Rudbeckia* spreads its seeds, most *R. laciniata* seeds have no specific microstructures, such as strong prickles or hooks, thus spreading to larger distances through exochory does not seem to be of great significance. *Rudbeckia* seeds are relatively heavy (~ 2.9 mg) and do not have structures enabling them to spread over long distances by wind^36,43,44^. Numerous reports indicate the linear distribution of *Rudbeckia* along roadsides and water streams^19,33,45,46^ and—considering that such places serve as natural migration corridors for many alien plants^28,47–49^—they can play a similar role in the case of *Rudbeckia*. The great abundance of *R. laciniata* in the wet meadows and river valleys is connected with the preference of this species for wet habitats (in SW Poland, for instance, *R. laciniata* grows on hundreds of localities in very dense patches and over great areas; Nobis 2021 pers. observations). The participation of hydrochory in spreading *R. laciniata* seeds is also important. A similar situation is found in the case of roadsides, where both increased air movement and the wheels of vehicles can facilitate the transport of *Rudbeckia* seeds over longer distances^33^.

The vertical distribution of seed banks in the soil may be connected with their longevity. The seeds of species creating transient seed banks are present only in surface soil whereas seeds of species creating short-term seed banks are present predominantly but not exclusively in the upper layer of soil^20^. In such banks the seeds live through only one germination season, usually, 16–18 months (transient), or they remain viable and germinable until at least the second germination season (short-term persistent)^50^. On the other hand, species forming long-term persistent soil banks, with the presence of the given species in the vegetation cover, are equally common in the upper and lower layers of the soil^20^, and the seeds should remain viable and germinable until at least the sixth germination season^50^. In our study, the vast majority of *Rudbeckia* seeds were detected in the upper layer of soil, what may suggest that *R. laciniata* forms rather short-term seed banks. The formation of a short-term seed bank by *Rudbeckia* was also reported by other authors^40,43^. This is important in the context of controlling invasive species, as regular mowing of *Rudbeckia* from above-ground vegetation should bring the desired results in a fairly short time.

The speed of seed germination is also an important and even crucial factor responsible for the invasive potential of a species^51,52^. Invasive plant species exhibit a greater ability to germinate early in comparison with other, native species. This suggests that the use of empty niches and avoiding competition in the early stages of a plant’s life can have greater significance than high competitive ability^51,52^. A comparison of species from the genus *Impatiens* indicated that the most problematic and invasive species in Europe, *Impatiens gladnulifera*, apart from quickly germinating, is also characterized by greater biomass of seedlings in comparison to both the aliens *I. parviflora* and *I. capensis*, as well as the native *I. noli-tangere*^53^. Thus it can be stated that the quick germination observed for *R. laciniata*, as well as the very large number of seedlings indicate a high predisposition to invasiveness, even taking into account the short-term seed bank formed by the taxon. Another invasive species of great productive potential, with also a short-term seed bank is *Heracleum mantegazzianum*^10^.

### The impact of *Rudbeckia laciniata* on the abundance and species composition of the soil seed bank

The presence of *R. laciniata* in the vegetation cover has a significant impact on the abundance of the soil seed bank, including a decrease of the diversity of species and an increase in the dominance index of the soil seed bank. On the other hand, a moderate cover of this species, even in the direct vicinity of highly invaded plots, did not negatively impact the parameters mentioned above, or this impact was small. A lack of impact on species richness, seed bank size and diversity (without considering invasive species) was also noted by Kundel et al.^12^ in the study on *Solidago canadensis* and *S. gigantea*. As a possible cause, the authors cite the small leaves of these species enabling the supply of new propagules, even when the density of goldenrod specimens is high. This could also be applicable to *R. laciniata*, the leaves of which, although larger than those of *Solidago* representatives, also do not form a dense canopy. During their research on the gradient of the *Bothriochloa* spp. invasion in the USA, Robertson and Hickman^24^ also noted a rapid decrease of species diversity and vegetation cover of the native flora, while the changes in the seed bank were seen only in an increase of the participation of *Bothriochloa* spp. seeds. The authors proposed the slower rate of changes in the soil seed bank as the main cause of this situation, stating that in the longer term, the diversity and density of the native seed banks will probably be smaller. Smaller changes in the soil seed bank in relation to those taking place in the vegetation cover were also observed after the invasion of *Miscanthus sacchariflorus*^23^ and *Euphorbia esula*^54^. There are also quite a few reports on the significant and quick decrease in the richness of species due to the invasion. In a meta-analysis based on a comparison of 58 pairs of invaded and non-invaded locations (for 18 various invasive species), Gioria et al.^55^ showed that in the case of the majority of taxa and habitats, the richness of species and the abundance of native seed banks were significantly lower in the invaded sites than the control plots.

In our study, the mean number of seeds and species was higher in the upper layer of soil than in the lower layer for all zones. Due to the small cover of other plant species in the invasion zones, the production of propagules was significantly smaller, which suggests a limited but rather constant flow of seeds from neighboring areas. An example can be the presence of such species as: *Stellaria graminea, Gnaphalium uliginosum, Erigeron annuus, Epilobium* sp., *Chenopodium polyspermum, Rumex* sp., *Juncus* sp., *Cerastium* sp., *Arabidopsis thaliana* in the upper layer of the soil seed bank of the invasion zone, but not detected in the above-ground vegetation in the zone. Another explanation for this phenomenon may be the relatively short time of the invasion. In such a case, seeds remaining in the invasion zone would still originate from the time *Rudbeckia* had a moderate density (beginning of the invasion) and did not die or reach the deeper layers of soil.

Besides *R. laciniata*, in all the zones and both examined meadows have a similar species composition, with a great abundance of *U. dioica*. The coexistence of *U. dioica* in the seed banks from sites occupied by invasive species has been recorded many times^12,55,56^. This species creates long lasting seed banks and has the ability to coexist for a long time in sites occupied by invaders^57^. It is also worth highlighting the high seed frequency of another invasive species, *Solidago canadensis*, which was found in the upper soil layer, although it was present with a much lower frequency in the vegetation zone. The effective propagation of *Solidago*, including its presence in the seed banks of non-invaded sites neighboring the invasion zone, was also found by Kundel et al.^12^ and Dölle and Wolfgang^58^.

### The relationship between the ground vegetation and soil seed bank

The similarity in the species composition, vegetation cover and soil seed bank in various plant communities show quite different results, indicating low^59^ or relatively high similarity^39,60,61^. Nevertheless, plant communities covered by invasive plants exhibit a high dissimilarity in the species composition of soil seed banks and ground vegetation^15,62^. In our case*,* the similarity between the composition of species in the ground vegetation and the content of the soil seed bank varied in subsequent invasion zones, however, the general trends in both locations were similar. The greatest dissimilarity occurred in the invasion zones, where the participation of species present only in the seed banks was the highest. Among the reasons discussed for the low similarity between the above-ground vegetation and soil seed banks may be the formation of a transition seed bank by some plants numerous in the ground vegetation, as well as the presence of species producing long-lasting seed banks that had accumulated in the soil and did not germinate due to the lack adequate conditions. Also, species that dominate in the vegetation can have various reproductive strategies, i.e. the advantage of vegetative reproduction. However, in the case of plant invasions, the time of the invasion seems to be the most significant^15^. On the other hand, in a situation where a reflection of the former vegetation has survived in the seed bank, as in the case of *R. laciniata*, we could suppose that removing the invader from the above-ground patches of vegetation may provide a good chance for regenerating the semi-native meadow communities.

## Supplementary Information

Supplementary Information.

## Data Availability

The datasets obtained during the current study are available from the corresponding author on reasonable request.
