# Supplementary material for: Effect of Rudbeckia laciniata invasion on soil seed banks of different types of meadow communities
Source: Sci Rep. 2022 Jun 29;12:10965. doi: 10.1038/s41598-022-14681-1 (PMC9242978; doi:10.1038/s41598-022-14681-1)
Supplement: Supplementary file 1 — Supplementary Information. [file 41598_2022_14681_MOESM1_ESM.pdf]

Supplementary material

Effect of *Rudbeckia laciniata* invasion on soil seed banks of different types of meadow communities

Elżbieta Jędrzejczak[1*], Ewelina Klichowska[1], Marcin Nobis[1*]

[1]Institute of Botany, Faculty of Biology, Jagiellonian University, Gronostajowa 3, 30-387 Kraków, Poland

Corresponding author: Elżbieta Jędrzejczak elzbieta.jedrzejczak@alumni.uj.edu.pl; Marcin Nobis m.nobis@uj.edu.pl

Table S1. The size of the soil seed bank in each zones in the fresh and in the wet meadow.

	Location	Fresh			Sum	Wet			Sum
	Zone	A	B	C		A	B	C	
1	*Achillea millefolium*	.	.	.	.	.	8	.	3
2	*Alchemilla* sp.	.	4	.	1	36	4	16	19
3	*Alnus incana*	.	.	.	.	36	.	.	12
4	*Angelica sylvestris*	8	36	96	47	8	.	.	3
5	*Anthoxanthum odoratum*	40	36	60	45	.	4	4	3
6	*Arabidopsis thaliana*	32	12	24	23	24	16	36	25
7	*Arenaria serpyllifolia*	.	.	4	1	4	.	.	1
8	*Armoracia rusticana*	.	16	.	5	.	.	.	.
9	*Betula pendula*	4	.	.	1	12	.	12	8
10	*Bromus* sp.	.	.	.	.	4	.	.	1
11	*Calamagrostis* sp.	.	8	8	5	.	8	16	8
12	*Cardamine pratensis*	.	4	12	5	.	.	.	.
13	*Cardaminopsis arenosa*	.	.	.	.	4	.	.	1
14	*Carex brizoides*	.	.	4	1	.	4	44	16
15	*Carex* sp.	8	.	.	3	.	8	40	16
16	*Carex spicata*	96	4	16	39	.	44	236	93
17	*Centaurea phrygia*	.	8	.	3	.	.	.	.
18	*Centaurea* sp.	.	12	.	4	.	.	.	.
19	*Cerastium* sp.	16	12	56	28	.	4	.	1
20	*Chenopodium album*	4	12	20	12	.	4	8	4
21	*Chenopodium polyspermum*	304	776	424	501	96	576	408	360
22	*Cirsium arvense*	8	4	8	7	4	16	40	20
23	*Cirsium oleraceum*	.	.	.	.	4	8	16	9
24	*Crepis biennis*	.	.	.	.	4	.	.	1
25	*Deschampsia cespitosa*	.	.	12	4	8	124	192	108
26	*Epilobium roseum*	.	.	8	3	.	.	4	1
27	*Epilobium* sp.	8	28	40	25	4	4	12	7
28	*Equisetum arvense*	.	.	.	.	.	.	4	1
29	*Erigeron annuus*	4	4	8	5	16	4	8	9

	Location	Fresh			Sum	Wet			Sum
	Zone	A	B	C		A	B	C	
30	*Erigeron canadensis*	.	.	8	3	.	4	.	1
31	*Fallopia convolvulus*	.	.	8	3	.	.	.	.
32	*Galeopsis* sp.	.	.	.	.	.	4	.	1
33	*Galeopsis speciosa*	4	.	.	1	4	4	4	4
34	*Galinsoga quadriradiata*	12	.	.	4	.	.	4	1
35	*Galium aparine*	16	.	.	5	56	52	16	41
36	*Galium mollugo*	4	12	16	11	.	4	.	1
37	*Geranium pusillum*	.	4	4	3	.	4	4	3
38	*Geranium* sp.	4	.	12	5	.	.	.	.
39	*Geum* sp.	.	.	4	1	.	.	.	.
40	*Glechoma hederacea*	4	.	4	3	.	.	.	.
41	*Gnaphalium uliginosum*	.	12	8	7	184	140	48	124
42	*Holcus lanatus*	164	220	1304	563	.	16	284	100
43	*Hypericum maculatum*	124	156	284	188	132	392	620	381
44	*Juncus bufonius*	16	36	60	37	84	4	4	31
45	*Juncus* sp.	.	16	28	15	640	876	244	587
46	*Juncus tenuis*	.	4	16	7	260	84	4	116
47	*Brassicaceae* SU	12	.	4	5	20	32	32	28
48	*Lamium purpureum*	8	.	4	4	.	.	20	7
49	*Luzula campestris*	.	.	4	1	.	40	120	53
50	*Lysimachia vulgaris*	.	.	.	.	20	96	16	44
51	*Mentha arvensis*	4	16	28	16	44	24	8	25
52	*Myosotis arvensis*	.	.	.	.	12	28	4	15
53	*Oxalis stricta*	148	112	136	132	120	56	28	68
54	*Persicaria hydropiper*	.	.	.	.	4	.	.	1
55	*Pimpinella saxifraga*	.	.	4	1	.	.	.	.
56	*Plantago lanceolata*	8	12	.	7	.	.	.	.
57	*Plantago major*	8	16	20	15	4	44	8	19
58	*Poaceae*	340	248	256	281	140	264	588	331
59	*Polygonum aviculare*	4	8	12	8	.	.	.	.
60	*Potentilla anserina*	.	.	.	.	.	.	8	3

Location		Fresh			Sum	Wet			Sum
Zone		**A**	**B**	**C**		**A**	**B**	**C**	
61	*Ranunculus acris*	.	4	.	1	.	.	.	.
62	*Ranunculus repens*	196	184	388	256	16	28	212	85
63	*Rorippa* sp.	.	.	.	.	20	8	.	9
64	*Rubus* sp.	20	20	.	13	40	8	4	17
65	*Rudbeckia laciniata*	4132	160	.	1431	17476	544	.	6007
66	*Rumex acetosa*	4	.	.	1	.	4	.	1
67	*Rumex obtusifolius*	40	12	8	20	304	44	20	123
68	*Rumex* sp.	48	.	4	17	.	.	.	.
69	*Sagina procumbens*	.	12	12	8	20	116	16	51
70	*Sambucus nigra*	.	.	.	.	4	4	.	3
71	*Saponaria officinalis*	.	.	.	.	12	.	.	4
72	*Scrophularia nodosa*	.	.	.	.	4	.	4	3
73	*Selinum carvifolia*	.	.	.	.	.	.	8	3
74	*Silene flos-cuculi*	.	.	28	9	4	20	4	9
75	*Solidago canadensis*	.	8	.	3	196	312	176	228
76	*Spergula arvensis*	.	.	.	.	.	4	.	1
77	*Stachys palustris*	.	.	.	.	.	48	16	21
78	*Stellaria graminea*	308	224	364	299	12	4	20	12
79	*Succisa pratensis*	.	.	.	.	.	4	4	3
80	*Taraxacum officinale* s. l.	.	.	.	.	4	.	.	1
81	*Trifolium pratense*	4	.	.	1	.	.	.	.
82	*Trifolium repens*	148	1024	44	405	8	48	8	21
83	*Urtica dioica*	1064	316	428	603	1008	1044	1284	1112
84	*Veronica arvensis*	4	.	.	1	.	.	.	.
85	*Veronica serpyllifolia*	76	100	176	117	.	4	4	3
86	*Veronica* sp.	80	20	32	44	.	8	60	23
87	*Vicia hirsuta*	20	.	.	7	12	16	4	11
88	*Viola arvensis*	4	12	32	16	.	4	.	1

The size of the soli seed bank – number of seeds/m^2; A – invasion zone, B – transition zone, C – control zone; SU – seedlings unidentified

Table S2. The number of samples (pots) in which plant species were recorded.

	Location	Fresh									Wet								
	Zone	A			B			C			A			B			C		
	Layer	U	L	Sum	U	L	Sum	U	L	Sum	U	L	Sum	U	L	Sum	U	L	Sum
1	*Achillea millefolium*	.	.	.	.	.	.	.	.	.	.	.	.	2	.	2	.	.	.
2	*Alchemilla* sp.	.	.	.	1	.	1	.	.	.	8	1	9	.	1	1	2	2	4
3	*Alnus incana*	.	.	.	.	.	.	.	.	.	6	.	6	.	.	.	.	.	.
4	*Angelica sylvestris*	2	.	2	7	.	7	13	1	14	2	.	2	.	.	.	.	.	.
5	*Anthoxanthum odoratum*	5	.	5	5	3	8	8	3	11	.	.	.	1	.	1	1	.	1
6	*Arabidopsis thaliana*	5	2	7	1	1	2	3	1	4	5	.	5	1	3	4	6	.	6
7	*Arenaria serpyllifolia*	.	.	.	.	.	.	.	1	1	1	.	1	.	.	.	.	.	.
8	*Armoracia rusticana*	.	.	.	2	1	3	.	.	.	.	.	.	.	.	.	.	.	.
9	*Betula pendula*	1	.	1	.	.	.	.	.	.	3	.	3	.	.	.	2	.	2
10	*Bromus* sp.	.	.	.	.	.	.	.	.	.	1	.	1	.	.	.	.	.	.
11	*Calamagrostis* sp.	.	.	.	2	.	2	1	.	1	.	.	.	2	.	2	2	1	3
12	*Cardamine pratensis*	.	.	.	1	.	1	3	.	3	.	.	.	.	.	.	.	.	.
13	*Cardaminopsis arenosa*	.	.	.	.	.	.	.	.	.	1	.	1	.	.	.	.	.	.
14	*Carex brizoides*	.	.	.	.	.	.	.	1	1	.	.	.	1	.	1	4	5	9
15	*Carex* sp.	2	.	2	.	.	.	.	.	.	.	.	.	1	1	2	4	1	5

	Location	Fresh									Wet								
	Zone	A			B			C			A			B			C		
	Layer	U	L	Sum	U	L	Sum	U	L	Sum	U	L	Sum	U	L	Sum	U	L	Sum
16	*Carex spicata*	8	8	16	1	.	1	2	1	3	.	.	.	7	2	9	14	3	17
17	*Centaurea phrygia*	.	.	.	2	.	2	.	.	.	.	.	.	.	.	.	.	.	.
18	*Centaurea* sp.	.	.	.	3	.	3	.	.	.	.	.	.	.	.	.	.	.	.
19	*Cerastium* sp.	4	.	4	2	.	2	4	5	9	.	.	.	1	.	1	.	.	.
20	*Chenopodium album*	1	.	1	2	1	3	.	5	5	.	.	.	1	.	1	.	2	2
21	*Chenopodium polyspermum*	14	12	26	18	20	38	15	14	29	6	10	16	16	19	35	16	18	34
22	*Cirsium arvense*	2	.	2	1	.	1	1	1	2	1	.	1	3	.	3	9	1	10
23	*Cirsium oleraceum*	.	.	.	.	.	.	.	.	.	.	1	1	2	.	2	4	.	4
24	*Crepis biennis*	.	.	.	.	.	.	.	.	.	1	.	1	.	.	.	.	.	.
25	*Deschampsia cespitosa*	.	.	.	.	.	.	3	.	3	2	.	2	12	4	16	14	6	20
26	*Epilobium roseum*	.	.	.	.	.	.	2	.	2	.	.	.	.	.	.	.	1	1
27	*Epilobium* sp.	2	.	2	6	.	6	7	.	7	1	.	1	1	.	1	3	.	3
28	*Equisetum arvense*	.	.	.	.	.	.	.	.	.	.	.	.	.	.	.	1	.	1
29	*Erigeron annuus*	1	.	1	1	.	1	1	.	1	2	2	4	1	.	1	2	.	2
30	*Erigeron canadensis*	.	.	.	.	.	.	2	.	2	.	.	.	.	1	1	.	.	.
31	*Fallopia convolvulus*	.	.	.	.	.	.	.	2	2	.	.	.	.	.	.	.	.	.

	Location	Fresh									Wet								
	Zone	A			B			C			A			B			C		
	Layer	U	L	Sum	U	L	Sum	U	L	Sum	U	L	Sum	U	L	Sum	U	L	Sum
32	*Galeopsis* sp.	.	.	.	.	.	.	.	.	.	.	.	.	1	.	1	.	.	.
33	*Galeopsis speciosa*	.	1	1	.	.	.	.	.	.	.	1	1	1	.	1	1	.	1
34	*Galinsoga quadriradiata*	3	.	3	.	.	.	.	.	.	.	.	.	.	.	.	.	1	1
35	*Galium aparine*	2	2	4	.	.	.	.	.	.	7	1	8	10	.	10	4	.	4
36	*Galium mollugo*	1	.	1	3	.	3	3	1	4	.	.	.	.	1	1	.	.	.
37	*Geranium pusillum*	.	.	.	1	.	1	1	.	1	.	.	.	.	1	1	.	1	1
38	*Geranium* sp.	1	.	1	.	.	.	.	3	3	.	.	.	.	.	.	.	.	.
39	*Geum* sp.	.	.	.	.	.	.	.	1	1	.	.	.	.	.	.	.	.	.
40	*Glechoma hederacea*	.	1	1	.	.	.	1	.	1	.	.	.	.	.	.	.	.	.
41	*Gnaphalium uliginosum*	.	.	.	2	1	3	2	.	2	8	12	20	8	16	24	4	5	9
42	*Holcus lanatus*	12	8	20	18	6	24	20	10	30	.	.	.	2	2	4	18	9	27
43	*Hypericum maculatum*	10	5	15	15	4	19	17	6	23	12	5	17	18	13	31	17	8	25
44	*Juncus bufonius*	4	.	4	5	3	8	9	3	12	.	4	4	.	1	1	1	.	1
45	*Juncus* sp.	.	.	.	4	.	4	5	.	5	17	19	36	20	20	40	14	15	29
46	*Juncus tenuis*	.	.	.	.	1	1	1	1	2	12	7	19	8	.	8	1	.	1
47	*Brassicaceae* SU	3	.	3	.	.	.	1	.	1	4	1	5	4	4	8	4	4	8

	Location	Fresh									Wet								
	**Zone**	**A**			**B**			**C**			**A**			**B**			**C**		
	**Layer**	**U**	**L**	**Sum**	**U**	**L**	**Sum**	**U**	**L**	**Sum**	**U**	**L**	**Sum**	**U**	**L**	**Sum**	**U**	**L**	**Sum**
48	*Lamium purpureum*	2	.	2	.	.	.	1	.	1	.	.	.	.	.	.	3	2	5
49	*Luzula campestris*	.	.	.	.	.	.	1	.	1	.	.	.	6	.	6	8	2	10
50	*Lysimachia vulgaris*	.	.	.	.	.	.	.	.	.	1	3	4	16	2	18	4	.	4
51	*Mentha arvensis*	1	.	1	3	.	3	4	.	4	3	4	7	4	1	5	.	2	2
52	*Myosotis arvensis*	.	.	.	.	.	.	.	.	.	2	1	3	6	1	7	1	.	1
53	SU	4	5	9	5	7	12	2	2	4	6	6	12	9	7	16	6	3	9
54	*Oxalis stricta*	10	10	20	13	7	20	12	13	25	10	11	21	4	9	13	4	2	6
55	*Persicaria hydropiper*	.	.	.	.	.	.	.	.	.	.	1	1	.	.	.	.	.	.
56	*Pimpinella saxifraga*	.	.	.	.	.	.	1	.	1	.	.	.	.	.	.	.	.	.
57	*Plantago lanceolata*	1	1	2	2	1	3	.	.	.	.	.	.	.	.	.	.	.	.
58	*Plantago major*	2	.	2	3	1	4	4	1	5	1	.	1	3	6	9	.	2	2
59	*Poaceae*	18	9	27	20	7	27	18	8	26	10	8	18	17	9	26	20	12	32
60	*Polygonum aviculare*	1	.	1	1	1	2	2	1	3	.	.	.	.	.	.	.	.	.
61	*Potentilla anserina*	.	.	.	.	.	.	.	.	.	.	.	.	.	.	.	1	1	2
62	*Ranunculus acris*	.	.	.	.	1	1	.	.	.	.	.	.	.	.	.	.	.	.
63	*Ranunculus repens*	17	5	22	19	4	23	20	11	31	3	1	4	6	1	7	12	12	24

	Location	Fresh									Wet								
	Zone	A			B			C			A			B			C		
	Layer	U	L	Sum	U	L	Sum	U	L	Sum	U	L	Sum	U	L	Sum	U	L	Sum
64	*Rorippa* sp.	.	.	.	.	.	.	.	.	.	3	1	4	.	2	2	.	.	.
65	*Rubus* sp.	2	3	5	5	.	5	.	.	.	6	2	8	1	1	2	1	.	1
66	*Rudbeckia laciniata*	20	15	35	12	4	16	.	.	.	20	20	40	20	5	25	.	.	.
67	*Rumex acetosa*	1	.	1	.	.	.	.	.	.	.	.	.	1	.	1	.	.	.
68	*Rumex obtusifolius*	6	2	8	3	.	3	2	.	2	16	11	27	7	.	7	4	.	4
69	*Rumex* sp.	6	.	6	.	.	.	1	.	1	.	.	.	.	.	.	.	.	.
70	*Sagina procumbens*	.	.	.	2	1	3	1	2	3	1	4	5	12	7	19	2	2	4
71	*Sambucus nigra*	.	.	.	.	.	.	.	.	.	1	.	1	1	.	1	.	.	.
72	*Saponaria officinalis*	.	.	.	.	.	.	.	.	.	3	.	3	.	.	.	.	.	.
73	*Scrophularia nodosa*	.	.	.	.	.	.	.	.	.	1	.	1	.	.	.	1	.	1
74	*Selinum carvifolia*	.	.	.	.	.	.	.	.	.	.	.	.	.	.	.	2	.	2
75	*Silene flos-cuculi*	.	.	.	.	.	.	5	1	6	.	1	1	4	1	5	.	1	1
76	*Solidago canadensis*	.	.	.	1	1	2	.	.	.	15	7	22	17	6	23	17	4	21
77	*Spergula arvensis*	.	.	.	.	.	.	.	.	.	.	.	.	.	1	1	.	.	.
78	*Stachys palustris*	.	.	.	.	.	.	.	.	.	.	.	.	8	.	8	3	.	3
79	*Stellaria graminea*	13	11	24	18	11	29	19	6	25	2	1	3	1	.	1	4	1	5

	Location	Fresh									Wet								
	Zone	A			B			C			A			B			C		
	Layer	U	L	Sum	U	L	Sum	U	L	Sum	U	L	Sum	U	L	Sum	U	L	Sum
80	*Succisa pratensis*	.	.	.	.	.	.	.	.	.	.	.	.	1	.	1	1	.	1
81	*Taraxacum officinale* s. l.	.	.	.	.	.	.	.	.	.	1	.	1	.	.	.	.	.	.
82	*Trifolium pratense*	1	.	1	.	.	.	.	.	.	.	.	.	.	.	.	.	.	.
83	*Trifolium repens*	3	17	20	20	2	22	6	3	9	1	1	2	7	5	12	1	1	2
84	*Urtica dioica*	20	13	33	20	9	29	20	11	31	17	9	26	20	17	37	20	19	39
85	*Veronica arvensis*	1	.	1	.	.	.	.	.	.	.	.	.	.	.	.	.	.	.
86	*Veronica serpyllifolia*	8	3	11	10	8	18	14	4	18	.	.	.	1	.	1	.	1	1
87	*Veronica* sp.	12	2	14	1	3	4	6	1	7	.	.	.	1	1	2	8	2	10
88	*Vicia hirsuta*	.	4	4	.	.	.	.	.	.	1	2	3	1	2	3	1	.	1
89	*Viola arvensis*	1	.	1	2	1	3	.	7	7	.	.	.	1	.	1	.	.	.

U - upper layer of soil 0-5 cm; L - lower layer of soil 6-10 cm; A – invasion zone, B – transition zone, C – control zone; SU – seedlings unidentified

Table S3. Similarity between the species composition of vegetation cover and soil seed bank based on the Sorensen index.

			Vegetation cover					
			Fresh			Wet		
			A	B	C	A	B	C
Seed bank	Fresh	A	0.4	-	-	-	-	-
		B	-	0.5	-	-	-	-
		C	-	-	0.4	-	-	-
	Wet	A	-	-	-	0.4	-	-
		B	-	-	-	-	0.5	-
		C	-	-	-	-	-	0.6

The range of the values of the Sorensen index: 0 – no similarity; 1 – full similarity; A – invasion zone, B – transition zone, C – control zone

Table S4. The taxa occurrence in soil seed bank and ground vegetation in the fresh and in the wet meadow.

	**Location**	**Fresh**						**Wet**					
	**Zone**	**A**		**B**		**C**		**A**		**B**		**C**	
	**Feature**	**Veg**	**Soil**	**Veg**	**Soil**	**Veg**	**Soil**	**Veg**	**Soil**	**Veg**	**Soil**	**Veg**	**Soil**
1	*Acer negundo*	.	.	.	.	.	.	.	.	+	.	.	.
2	*Acer pseudoplatanus*	+	.	+	.	+	.	.	.	+	.	+	.
3	*Achillea millefolium*	+	.	+	.	+	.	.	.	+	+	+	.
4	*Aegopodium podagraria*	+	.	.	.	.	.	+	.	+	.	+	.
5	*Agrimonia eupatoria*	.	.	.	.	+	.	.	.	.	.	.	.
6	*Agrostis capillarias*	+	.	+	.	+	.	+	.	+	.	+	.
7	*Agrostis gigantea*	+	.	+	.	+	.	.	.	+	.	+	.
8	*Agrostis* sp.	.	.	.	.	.	.	+	.	.	.	.	.
9	*Alchemilla* sp.	.	.	.	+	.	.	.	+	+	+	+	+
10	*Alnus glutinosa*	.	.	.	.	.	.	.	.	+	.	.	.

	Location	Fresh						Wet					
	Zone	A		B		C		A		B		C	
	Feature	Veg	Soil	Veg	Soil	Veg	Soil	Veg	Soil	Veg	Soil	Veg	Soil
11	*Alnus incana*	.	.	.	.	.	.	.	+	+	.	.	.
12	*Alopecurus pratensis*	.	.	.	.	+	.	+	.	+	.	+	.
13	*Angelica sylvestris*	+	+	+	+	+	+	+	+	+	.	+	.
14	*Anthoxanthum odoratum*	.	+	+	+	+	+	.	.	+	+	+	+
15	*Arabidopsis thaliana*	.	+	.	+	.	+	.	+	.	+	.	+
16	*Arenaria serpyllifolia*	.	.	.	.	.	+	.	+	.	.	.	.
17	*Armoracia rusticana*	.	.	.	+	.	.	.	.	.	.	+	.
18	*Arrhenatherum elatius*	+	.	+	.	+	.	.	.	+	.	+	.
19	*Artemisia vulgaris*	.	.	.	.	.	.	.	.	+	.	.	.
20	*Betula pendula*	.	+	+	.	+	.	.	+	+	.	+	+
21	*Bromus* sp.	.	.	.	.	.	.	.	+	.	.	.	.
22	*Calamagrostis arundinacea*	.	.	+	.	.	.	.	.	.	.	.	.
23	*Calamagrostis epigejos*	.	.	.	.	+	.	+	.	+	.	+	.
24	*Calamagrostis* sp.	.	.	.	+	.	+	.	.	.	+	.	+
25	*Campanula patula*	.	.	+	.	+	.	.	.	+	.	+	.
26	*Cardamine pratensis*	.	.	.	+	.	+	.	.	.	.	.	.
27	*Cardaminopsis arenosa*	.	.	.	.	.	.	.	+	.	.	.	.
28	*Carex brizoides*	.	.	.	.	+	+	+	.	+	+	+	+
29	*Carex hirta*	.	.	+	.	+	.	+	.	+	.	+	.
30	*Carex muricata* agg.	.	.	.	.	.	.	.	.	.	.	+	.
31	*Carex pallescens*	.	.	.	.	.	.	.	.	+	.	.	.
32	*Carex* sp.	.	+	.	.	.	.	+	.	+	+	.	+
33	*Carex spicata*	.	+	.	+	.	+	.	.	.	+	.	+
34	*Carpinus betulus*	+	.	.	.	.	.	.	.	.	.	.	.
35	*Centaurea jacea*	+	.	+	.	+	.	.	.	+	.	+	.
36	*Centaurea phrygia*	.	.	+	+	.	.	.	.	.	.	.	.
37	*Centaurea* sp.	.	.	.	+	.	.	.	.	.	.	.	.
38	*Cerastium glomeratum*	.	.	.	.	.	.	.	.	+	.	.	.
39	*Cerastium* sp.	.	+	.	+	.	+	.	.	.	+	.	.

	Location	Fresh						Wet					
	Zone	A		B		C		A		B		C	
	Feature	Veg	Soil	Veg	Soil	Veg	Soil	Veg	Soil	Veg	Soil	Veg	Soil
40	*Chaerophyllum aromaticum*	+	.	.	.	.	.	.	.	+	.	+	.
41	*Chenopodium album*	.	+	.	+	.	+	.	.	.	+	.	+
42	*Chenopodium polyspermum*	.	+	+	+	.	+	.	+	.	+	.	+
43	*Cirsium arvense*	+	+	+	+	+	+	.	+	+	+	+	+
44	*Cirsium oleraceum*	.	.	.	.	.	.	+	+	+	+	+	+
45	*Cirsium vulgare*	.	.	.	.	+	.	.	.	.	.	.	.
46	*Clinopodium vulgare*	.	.	.	.	+	.	.	.	.	.	.	.
47	*Convolvulus arvensis*	+	.	+	.	+	.	.	.	+	.	+	.
48	*Corylus avellana*	.	.	.	.	.	.	+	.	.	.	.	.
49	*Crepis biennis*	.	.	+	.	.	.	.	+	.	.	+	.
50	*Cruciata glabra*	+	.	.	.	.	.	.	.	.	.	+	.
51	*Dactylis glomerata*	.	.	+	.	+	.	.	.	+	.	+	.
52	*Daucus carota*	.	.	.	.	+	.	.	.	.	.	.	.
53	*Deschampsia cespitosa*	.	.	.	.	+	+	.	+	+	+	+	+
54	*Elymus repens*	+	.	+	.	+	.	.	.	+	.	+	.
55	*Epilobium ciliatum*	.	.	+	.	+	.	.	.	+	.	.	.
56	*Epilobium hirsutum*	.	.	+	.	.	.	.	.	.	.	.	.
57	*Epilobium roseum*	.	.	.	.	.	+	.	.	.	.	.	+
58	*Epilobium* sp.	.	+	.	+	.	+	.	+	.	+	.	+
59	*Epilobium tetragonum*	.	.	.	.	.	.	.	.	.	.	+	.
60	*Equisetum arvense*	+	.	+	.	+	.	+	.	+	.	+	.
61	*Equisetum palustre*	.	.	.	.	.	.	+	.	+	.	+	.
62	*Equisetum sylvaticum*	.	.	.	.	+	.	.	.	.	.	.	.
63	*Equisetum telmateia*	+	.	.	.	.	.	.	.	.	.	.	.
64	*Erigeron annuus*	.	+	+	+	.	+	.	+	.	+	+	+
65	*Erigeron canadensis*	.	.	.	.	.	+	.	.	.	+	.	.
66	*Euphorbia cyparissias*	.	.	.	.	+	.	.	.	+	.	+	.
67	*Fallopia convolvulus*	+	.	+	.	+	+	.	.	.	.	.	.
68	*Festuca arundinacea*	.	.	.	.	.	.	.	.	+	.	.	.

	Location	Fresh						Wet					
	Zone	A		B		C		A		B		C	
	Feature	Veg	Soil	Veg	Soil	Veg	Soil	Veg	Soil	Veg	Soil	Veg	Soil
69	*Festuca gigantea*	+	.	.	.	.	.	+	.	.	.	.	.
70	*Festuca pratensis*	.	.	+	.	+	.	.	.	+	.	+	.
71	*Festuca rubra*	.	.	+	.	+	.	.	.	+	.	+	.
72	*Festuca* sp.	.	.	.	.	.	.	.	.	.	.	+	.
73	*Filipendula ulmaria*	.	.	.	.	.	.	.	.	+	.	+	.
74	*Fragaria vesca*	.	.	.	.	.	.	.	.	.	.	+	.
75	*Frangula alnus*	.	.	.	.	.	.	.	.	+	.	+	.
76	*Galeopsis* sp.	.	.	.	.	.	.	.	.	.	+	.	.
77	*Galeopsis speciosa*	+	+	+	.	+	.	+	+	+	+	+	+
78	*Galinsoga quadriradiata*	.	+	.	.	.	.	.	.	.	.	.	+
79	*Galium aparine*	+	+	.	.	+	.	+	+	+	+	+	+
80	*Galium mollugo*	+	+	+	+	+	+	.	.	.	+	.	.
81	*Galium verum*	.	.	.	.	+	.	.	.	.	.	.	.
82	*Geranium palustre*	.	.	.	.	.	.	+	.	+	.	+	.
83	*Geranium pusillum*	.	.	.	+	.	+	.	.	.	+	.	+
84	*Geranium* sp.	.	+	.	.	.	+	.	.	.	.	.	.
85	*Geum* sp.	.	.	.	.	.	+	.	.	.	.	.	.
86	*Geum urbanum*	+	.	+	.	.	.	.	.	.	.	.	.
87	*Glechoma hederacea*	+	+	+	.	+	+	.	.	+	.	+	.
88	*Gnaphalium uliginosum*	.	.	.	+	.	+	.	+	.	+	.	+
89	*Heracleum sphondylium*	.	.	+	.	+	.	.	.	+	.	+	.
90	*Hieracium lachenalii*	.	.	.	.	+	.	.	.	.	.	.	.
91	*Hieracium umbellatum*	.	.	.	.	.	.	.	.	+	.	.	.
92	*Holcus lanatus*	+	+	+	+	+	+	+	.	+	+	+	+
93	*Holcus mollis*	.	.	+	.	+	.	+	.	+	.	+	.
94	*Humulus lupulus*	.	.	.	.	.	.	.	.	+	.	+	.
95	*Hypericum maculatum*	+	+	+	+	+	+	+	+	+	+	+	+
96	*Juncus bufonius*	.	+	.	+	.	+	.	+	.	+	.	+
97	*Juncus effusus*	.	.	.	.	+	.	+	.	+	.	+	.

	Location	Fresh						Wet					
	**Zone**	**A**		**B**		**C**		**A**		**B**		**C**	
	**Feature**	**Veg**	**Soil**	**Veg**	**Soil**	**Veg**	**Soil**	**Veg**	**Soil**	**Veg**	**Soil**	**Veg**	**Soil**
98	*Juncus* sp.	.	.	.	+	.	+	.	+	.	+	.	+
99	*Juncus tenuis*	.	.	.	+	.	+	.	+	+	+	.	+
100	*Brassicaceae*	.	+	.	.	.	+	.	+	.	+	.	+
101	*Lamium album*	.	.	.	.	+	.	.	.	.	.	.	.
102	*Lamium purpureum*	.	+	.	.	.	+	.	.	.	.	+	+
103	*Lathyrus pratensis*	.	.	.	.	.	.	.	.	+	.	+	.
104	*Lotus pedunculatus*	.	.	.	.	.	.	.	.	+	.	.	.
105	*Luzula campestris*	.	.	.	.	+	+	.	.	+	+	+	+
106	*Lysimachia nummularia*	.	.	+	.	+	.	+	.	+	.	+	.
107	*Lysimachia vulgaris*	.	.	.	.	+	.	+	+	+	+	+	+
108	*Malus* sp. (seedling)	.	.	+	.	.	.	.	.	.	.	.	.
109	*Mentha arvensis*	.	+	+	+	+	+	.	+	+	+	+	+
110	*Mentha longifolia*	.	.	+	.	+	.	.	.	.	.	.	.
111	*Myosotis arvensis*	.	.	.	.	.	.	.	+	.	+	+	+
112	*Oxalis stricta*	+	+	.	+	.	+	.	+	+	+	.	+
113	*Persicaria hydropiper*	.	.	.	.	.	.	.	+	.	.	.	.
114	*Phleum pratense*	.	.	+	.	+	.	.	.	+	.	+	.
115	*Pimpinella saxifraga*	+	.	+	.	+	+	.	.	+	.	+	.
116	*Pinus sylvestris* (seedling)	.	.	+	.	.	.	.	.	.	.	.	.
117	*Plantago lanceolata*	.	+	.	+	+	.	.	.	+	.	.	.
118	*Plantago major*	.	+	+	+	.	+	.	+	.	+	.	+
119	*Platanthera bifolia*	.	.	.	.	.	.	.	.	+	.	.	.
120	*Poa palustris*	.	.	.	.	.	.	.	.	+	.	+	.
121	*Poa pratensis*	.	.	+	.	+	.	+	.	+	.	+	.
122	*Poa trivialis*	.	.	+	.	.	.	.	.	.	.	.	.
123	*Poaceae*	+	+	.	+	.	+	+	+	.	+	.	+
124	*Polygonum aviculare*	.	+	.	+	.	+	.	.	.	.	.	.
125	*Populus* sp.	.	.	.	.	.	.	.	.	+	.	.	.
126	*Populus tremula*	.	.	.	.	.	.	.	.	.	.	+	.

	Location	Fresh						Wet					
	**Zone**	**A**		**B**		**C**		**A**		**B**		**C**	
	**Feature**	**Veg**	**Soil**	**Veg**	**Soil**	**Veg**	**Soil**	**Veg**	**Soil**	**Veg**	**Soil**	**Veg**	**Soil**
127	*Potentilla anserina*	.	.	.	.	.	.	.	.	.	.	+	+
128	*Potentilla erecta*	.	.	.	.	.	.	.	.	+	.	.	.
129	*Prunus spinosa*	.	.	.	.	+	.	.	.	.	.	.	.
130	*Pteridium aquilinum*	+	.	+	.	.	.	.	.	.	.	.	.
131	*Pulmonaria obscura*	.	.	.	.	.	.	+	.	.	.	.	.
132	*Quercus robur*	.	.	+	.	.	.	.	.	+	.	+	.
133	*Ranunculus acris*	.	.	+	+	+	.	.	.	.	.	.	.
134	*Ranunculus repens*	.	+	+	+	+	+	+	+	+	+	+	+
135	*Rorippa palustris*	.	.	.	.	+	.	.	.	.	.	.	.
136	*Rosa* sp.	.	.	+	.	+	.	.	+	.	+	.	.
137	*Rubus glivicensis*	+	.	+	.	+	.	.	.	+	.	.	.
138	*Rubus idaeus*	.	.	.	.	.	.	+	.	+	.	+	.
139	*Rubus* sp.	.	+	.	+	.	.	.	+	.	+	.	+
140	*Rudbeckia laciniata*	+	+	+	+	.	.	+	+	+	+	.	.
141	*Rumex acetosa*	.	+	+	.	+	.	.	.	+	+	.	.
142	*Rumex acetosella*	.	.	.	.	.	.	.	.	.	.	+	.
143	*Rumex crispus*	.	.	.	.	.	.	.	.	.	.	+	.
144	*Rumex obtusifolius*	.	+	.	+	.	+	.	+	.	+	.	+
145	*Rumex* sp.	.	+	.	.	.	+	.	.	.	.	.	.
146	*Sagina procumbens*	.	.	.	+	.	+	.	+	.	+	.	+
147	*Sambucus nigra*	.	.	.	.	.	.	.	+	.	+	.	.
148	*Sanguisorba officinalis*	.	.	.	.	.	.	+	.	+	.	+	.
149	*Saponaria officinalis*	.	.	.	.	.	.	.	+	.	.	.	.
150	*Scirpus sylvaticus*	.	.	.	.	.	.	.	.	.	.	+	.
151	*Scrophularia nodosa*	.	.	.	.	.	.	.	+	.	.	.	+
152	*Selinum carvifolia*	+	.	+	.	+	.	.	.	+	.	+	+
153	*Senecio nemorensis*	.	.	+	.	+	.	.	.	.	.	.	.
154	*Silene flos-cuculi*	.	.	+	.	+	+	.	+	+	+	+	+
155	*Solidago canadensis*	.	.	+	+	+	.	+	+	+	+	+	+

	Location	Fresh						Wet					
	**Zone**	**A**		**B**		**C**		**A**		**B**		**C**	
	**Feature**	**Veg**	**Soil**	**Veg**	**Soil**	**Veg**	**Soil**	**Veg**	**Soil**	**Veg**	**Soil**	**Veg**	**Soil**
156	*Solidago gigantea*	.	.	.	.	.	.	.	.	+	.	.	.
157	*Solidago virgaurea*	.	.	+	.	+	.	.	.	.	.	.	.
158	*Spergula arvensis*	.	.	.	.	.	.	.	.	.	+	.	.
159	*Stachys officinalis*	.	.	.	.	+	.	.	.	.	.	.	.
160	*Stachys palustris*	.	.	.	.	+	.	.	.	+	+	.	+
161	*Stellaria graminea*	.	+	+	+	+	+	.	+	+	+	+	+
162	*Stellaria holostea*	.	.	.	.	.	.	.	.	+	.	.	.
163	*Succisa pratensis*	.	.	.	.	.	.	.	.	.	+	.	+
164	*Tanacetum vulgare*	.	.	+	.	.	.	.	.	.	.	.	.
165	*Taraxacum officinale* s. l.	.	.	.	.	.	.	.	+	.	.	.	.
166	*Tilia cordata*	+	.	.	.	.	.	.	.	.	.	.	.
167	*Torilis japonica*	.	.	.	.	+	.	.	.	+	.	.	.
168	*Tragopogon orientalis*	.	.	.	.	.	.	.	.	+	.	+	.
169	*Trifolium pratense*	.	+	.	.	.	.	.	.	.	.	.	.
170	*Trifolium repens*	.	+	+	+	.	+	.	+	.	+	+	+
171	*Ulmus* sp.	.	.	.	.	.	.	+	.	.	.	.	.
172	*Urtica dioica*	+	+	+	+	+	+	+	+	+	+	+	+
173	*Veronica chamaedrys*	+	+	+	.	+	.	.	.	+	.	+	.
174	*Veronica montana*	.	.	.	.	.	.	.	.	.	.	+	.
175	*Veronica serpyllifolia*	.	+	.	+	.	+	.	.	.	+	.	+
176	*Veronica* sp.	.	+	.	+	.	+	.	.	.	+	.	+
177	*Vicia cracca*	.	.	.	.	+	.	.	.	+	.	+	.
178	*Vicia hirsuta*	+	+	.	.	+	.	.	+	+	+	+	+
179	*Vicia sativa* subsp. *nigra*	.	.	.	.	.	.	.	.	+	.	.	.
180	*Vicia sepium*	.	.	.	.	.	.	.	.	+	.	+	.
181	*Vicia* sp.	+	.	.	.	.	.	.	.	.	.	+	.
182	*Vicia tetrasperma*	.	.	.	.	.	.	.	.	+	.	+	.
183	*Viola arvensis*	.	+	.	+	+	+	.	.	.	+	.	.

A – invasion zone, B – transition zone, C – control zone, Veg – ground vegetation; Soil – soil seed bank
